# HOW DO GREEN CARE FARMS FOR PEOPLE LIVING WITH DEMENTIA SUCCESSFULLY INTEGRATE OUTDOOR SPACES?

**DOI:** 10.1093/geroni/igac059.1293

**Published:** 2022-12-20

**Authors:** Katharina Rosteius, Bram de Boer, Hilde Verbeek

**Affiliations:** Maastricht University, Maastricht, Limburg, Netherlands; Maastricht University, Maastricht, Limburg, Netherlands; Maastricht University, Maastricht, Limburg, Netherlands

## Abstract

Green Care Farms (GCFs) are an innovative care environment for vulnerable groups. This study explores the design, and the successful integration of the natural environments of GCFs. By actively integrating outdoor spaces into daily care, they may encourage residents to participate in meaningful activities. A case study was conducted on a Dutch GCF using mixed ethnographic methods. 129 hours of observations of daily life were combined with 24 semi-structured interviews with residents, family, volunteers, staff and management, and a focus group with staff. The physical environment was assessed quantitatively. Data was analyzed thematically and triangulated. Results indicate that some factors supported the integration of the outdoor spaces into daily care. First, the physical environment was specifically designed to stimulate residents. Second, the outdoors was easily and openly accessible for residents. Third, residents’ security was supported in several ways. Lastly, staff members creatively motivated residents to use the outside spaces.

